# Predicting bilirubin trends in preterm infants using patient specific exponential decay model

**DOI:** 10.1038/s41390-025-03808-5

**Published:** 2025-01-13

**Authors:** Manoj Biniwale

**Affiliations:** Cedars Sinai Medical Center, Los Angeles, CA USA

Jaundice is one of the most common problems faced by preterm infants in the first month of life. Decreased life span of red blood cells, immaturity of liver enzymes, increased enterohepatic circulation, and associated illnesses such as sepsis and hemolysis play important role in contributing towards increased bilirubin levels in preterm infants. Other factors including delayed initiation of enteral feeding, unable to tolerate feeds and bruising at the time of delivery may impose additional risk of developing hyperbilirubinemia in these infants.^1^ Unlike in term infants, hyperbilirubinemia is more protracted and largely unpredictable in preterm infants. Chronic bilirubin encephalopathy may manifest as kernicterus leading to choreoathetoid cerebral palsy, hearing loss or sensorimotor integration disorders. Even though exact serum bilirubin levels causing acute bilirubin encephalopathy are not known, the preterm infants despite rigorous monitoring are at higher risk for kernicterus at lower bilirubin concentrations than term infants.^2^

Bilirubin research in preterm infants is primarily targeted towards early detection, determination of the treatment thresholds to prevent encephalopathy and assessment of neurological consequences. Current guidelines typically focus on initial management of hyperbilirubinemia in preterm infants to prevent kernicterus. Risk for prolonged and life-threatening jaundice remains in the preterm infants beyond first week of life which can lead to undesirable consequences.^3^ Gestational age and hour of life specific nomograms for preterm infants have been created to help develop early management decisions.^4^ A recent study has demonstrated use of an electronic clinical decision-making tool to alert physicians of critical values of bilirubin levels in preterm infants.^5^ While critical hyperbilirubinemia is diagnosed in early phase there is scarcity in the literature on natural course of bilirubin levels at various gestational ages beyond first week of life. Also, the factors indirectly affecting bilirubin concentrations including inflammatory makers such as c reactive protein (CRP) or necrotizing enterocolitis (NEC) have rarely been taken into consideration for predicting bilirubin levels especially in preterm infants.

The article by Chen et al. in this issue of Pediatric Research highlights methods to characterize serum bilirubin dynamics in preterm infants (24–32 weeks gestation) using patient-specific exponential decay model using computer-assisted diagnostic tool.^6^ The study model describes how to predict trends of bilirubin from day 3-4 to first weeks of life to optimize monitoring in neonatal intensive care unit and estimate high risk events affecting bilirubin levels. This prospective study was a part of originally collected data to develop a computer assisted diagnostic tool by analyzing cardiorespiratory signals in the real time for diagnosing infection risk. Cohort of 24–32 week preterm infants was studied over 6 years span. Infants with at least 4 values of serum bilirubin levels were included. To effectively characterize natural evolution, the infants with infrequent values as well as values of bilirubin while receiving phototherapy or exchange transfusion were excluded. Relevant clinical parameters such as demographic factors, maternal and infants’ laboratory tests were fed into the model to analyze effects on the serum bilirubin concentrations.

Decay models are applicable in datasets to describe systematic decrease in quantity over time as determined by a mathematical function. Exponential decay model refers to an equation measuring decrease in proportion to current value over time.^7^ Compared to linear regression model, the exponential decay model performs better when the rate of change is not constant but proportional to the current values. As these models require multiple time points for fitting to have a valid trend, the authors rightfully excluded patients with fewer than 4 values. Bilirubin values measured after treatment with phototherapy or exchange transfusion may disrupt the natural progression in preterm infants and may not fit into the model to describe natural rate of rise or fall. Patient specific exponential decay models were developed to account for individual variability while adjusting for gestational age and postnatal age. To analyze the model for assessing clinical outcomes first histograms were created. Parameters for central tendency such as mean and range from the histograms were useful in assessing variability and consistency as well as performance of the model. Further, root mean square error (RMSE) was used to assess differences between predicted value and actual value. RMSE was primarily used to understand the effects of risk events such as NEC or change in inflammatory markers such as CRP. Lastly the authors assessed impact of individual parameters on the outcomes of the median model using sensitivity analyses. This stepwise approach shows a robust study design in the model creation.

This study was performed on 72 preterm infants born between gestational age of 24 2/7 weeks and 31 6/7 weeks from multiple institutions with each of the infants had 4–20 measurements of serum bilirubin concentrations. The results of the study showed that general models including linear regression and exponential decay with larger inter-individual variability necessitating patient specific modeling for further analyses. The rates of decay were noted to be affected in the individual patient graphs by NEC as well as CRP as demonstrated by modeling errors with high RMSE values. These findings are very important from clinical standpoint as various factors play roles in affecting bilirubin values besides natural progression. If the bilirubin levels are higher than predictable decay and not following the curve it could be either related to other comorbidities or a subtle sign of developing early infection, NEC, or other complications. Two significant factors identified through sensitivity analyses were gestational age and compensatory factor to time shift representing varying maturity levels at same gestational age.

This is one of the unique studies which exponential decay model to analyze the trajectory of serum bilirubin levels in preterm infants. Overall, this study highlights the importance of bilirubin trending in health and disease for preterm infants beyond first week of life. The advantages of using exponential decay model include its simplicity to use through mathematical formula, accuracy of capturing decrease in values, wide range of applications and easy recognition through graphs.^7^ Researchers must use caution using the model as demonstrated in this manuscript as accuracy of the model is affected by various clinical parameters such as gestational age and clinical illness. In these situations, patient specific models provide better interpretation of the data. RMSE measures average magnitude of errors between predicted and observed values. Lower RMSE values indicate predictive values closer to actual values and give better fit. High RMSE values indicate poor prediction as in this manuscript indicating significant events such as NEC or CRP affecting the decrease in bilirubin levels. Another way of measuring errors in observations compared to predicted values is using mean absolute error (MAE) which has been used in the past to study serum bilirubin prediction.^8^ RMSE is more sensitive than MAE addressing larger errors.

This study is based on relatively small population of patients. Studies using more comprehensive data from various large institutions across the world may be able to validate and form algorithms for more accurate bilirubin detection and predict future therapies. Additional factors that could be potentially studied in measuring trend of bilirubin levels in preterm infants include racial distribution, genomic variability, breast milk feedings, and use of medications. There are number of studies related to population of preterm infants in which researchers can potentially apply this methodology of creating patient specific exponential decay. Studies related to transcutaneous bilirubin compared to serum bilirubin in term and preterm infants could be tested with decay model with various factors assessed using RMSE. Additional areas of research using similar model may include studies related to ventilation support, oxygen needs, hemoglobin levels as well as hemodynamic studies for patent ductus arteriosus, pulmonary vascular resistance etc. Studying multiple parameters will lead to a better understanding of trends in managing preterm infants as well as better prediction in extent of comorbidities. With advancement of artificial intelligence and various machine learning models, the future management of infants will be streamlined with decrease in physician or center related variabilities ultimately leading to better outcomes.
